# The supporting role of Visual Evoked Potentials for the diagnosis of Optic Neuritis within the 2022 ICON criteria

**DOI:** 10.1177/13524585261424125

**Published:** 2026-03-30

**Authors:** Giacomo Greco, Eleonora Rigoni, Francesco Masi, Giuseppe Cosentino, Massimiliano Todisco, Lara Ahmad, Eleonora Tavazzi, Eduardo Caverzasi, Anna Pichiecchio, Stefania Bianchi Marzoli, Michele Terzaghi, Elena Colombo, Matteo Gastaldi

**Affiliations:** Department of Brain and Behavioural Sciences, University of Pavia, Pavia, Italy; Multiple Sclerosis Center, IRCCS Mondino Foundation, Pavia, Italy; Multiple Sclerosis Center, IRCCS Mondino Foundation, Pavia, Italy; Multiple Sclerosis Center, IRCCS Mondino Foundation, Pavia, Italy; Department of Brain and Behavioural Sciences, University of Pavia, Pavia, Italy; Neurophysiology Unit, IRCCS Mondino Foundation, Pavia, Italy; Neurophysiology Unit, IRCCS Mondino Foundation, Pavia, Italy; Multiple Sclerosis Center, IRCCS Mondino Foundation, Pavia, Italy; Multiple Sclerosis Center, IRCCS Mondino Foundation, Pavia, Italy; Advanced Imaging and Artificial Intelligence Center, Department of Neuroradiology, IRCCS Mondino Foundation, Pavia, Italy; Department of Brain and Behavioural Sciences, University of Pavia, Pavia, Italy; Advanced Imaging and Artificial Intelligence Center, Department of Neuroradiology, IRCCS Mondino Foundation, Pavia, Italy; Neuro-Ophthalmology Center and Ocular Electrophysiology Laboratory, IRCCS Istituto Auxologico Italiano IRCCS Capitanio Hospital, Milan, Italy; Department of Brain and Behavioural Sciences, University of Pavia, Pavia, Italy; Neurophysiology Unit, IRCCS Mondino Foundation, Pavia, Italy; Multiple Sclerosis Center, IRCCS Mondino Foundation, Pavia, Italy; Neuroimmunology Laboratory, IRCCS Mondino Foundation, Pavia, Italy

**Keywords:** Optic neuritis, diagnostic criteria, visual evoked potentials, optical coherence tomography, MRI

## Abstract

**Background:**

Visual evoked potentials (VEPs) are commonly used in the assessment of optic neuritis (ON) but were not included in the International Consortium for ON (ICON) 2022 diagnostic criteria as supporting tests.

**Objectives:**

We aimed to assess VEPs diagnostic performances and their addition to the ICON criteria in a large consecutive cohort of patients with suspected ON.

**Methods:**

We screened 207 patients with suspected ON and included 71 who performed MRI, OCT, and VEPs within 30 days from symptoms onset. We calculated diagnostic performances for each test and for the ICON 2022 criteria with and without the addition of VEPs.

**Results:**

VEPs had high overall accuracy (88.7%; 95% confidence interval [CI]: 79.0–95.0) and area under the curve in the ROC analysis (0.89, CI: 0.82–0.97]). The addition of VEPs to the ICON 2022 criteria led to an improvement in sensitivity (100.0%, CI: 92.5–100.0), PPV (88.7%, CI: 79.6–94.0) and NPV (100%, CI: 78.2–100.0), maintaining the same specificity (75.0%, CI: 53.2–90.2), improving diagnostic accuracy to 91.5% (CI: 82.5–96.8).

**Conclusions:**

VEPs are reliable for the assessment of suspected ON, and they could be considered as a further paraclinical test in the ICON criteria.

## Introduction

Optic neuritis (ON) is an inflammatory disorder of the optic nerve, which presents with subacute vision loss, retro-orbital pain that worsens on eye movements and dyschromatopsia.^1^ It is one of the most common manifestations of CNS demyelinating disorders including multiple sclerosis (MS), myelin oligodendrocyte glycoprotein (MOG) antibody-associated disorder (MOGAD), and neuromyelitis optica spectrum disorder (NMOSD) with or without aquaporin-4-IgG (AQP4-IgG).^2–5^ Early diagnosis of ON in these instances is crucial to start symptomatic and long-term treatment. However, misdiagnosis rate can be as high as 60% of patients initially framed as ON.^6^

Recently, the International Consortium on Optic Neuritis (ICON) has proposed a set of diagnostic criteria to provide clear guidelines for ON assessment.^7^ They consist of a combination of clinical features and paraclinical tests to help acute ON assessment. Clinical criteria include monocular, subacute loss of vision associated with retro-orbital pain, dyschromatopsia, and a relative afferent pupillary deficit (RAPD). In addition, at least one positive paraclinical test is required, either optical coherence tomography (OCT) intereye asymmetries or evidence of optic disk swelling, MRI lesions (either contrast-enhancing or hyperintense signal in the optic nerve), and biological features such as serum antibodies to AQP4 or MOG, or CSF oligoclonal bands (OCBs).^7^

Visual evoked potentials (VEPs) are a neurophysiological technique which is commonly used in the diagnostic assessment of ON.^8,9^ Specific VEP alterations, such as a delayed latency of the P100 wave peak time or its asymmetry compared with the contralateral eye,^10^ are highly specific for ON.^11,12^ Despite this, VEPs were not included among supportive paraclinical tests in the 2022 ICON criteria, since they were considered non-specific by 52% of the experts in the panel.^7^

The aim of this study was to assess the diagnostic performance of VEPs in the workup of a suspected ON, analyzing a large consecutive cohort of patients who performed all VEPs, MRI, and OCT, besides clinical assessment, and to compare the respective diagnostic performance measures.

## Materials and methods

### Inclusion and exclusion criteria and ON diagnosis

**Figure 1.**
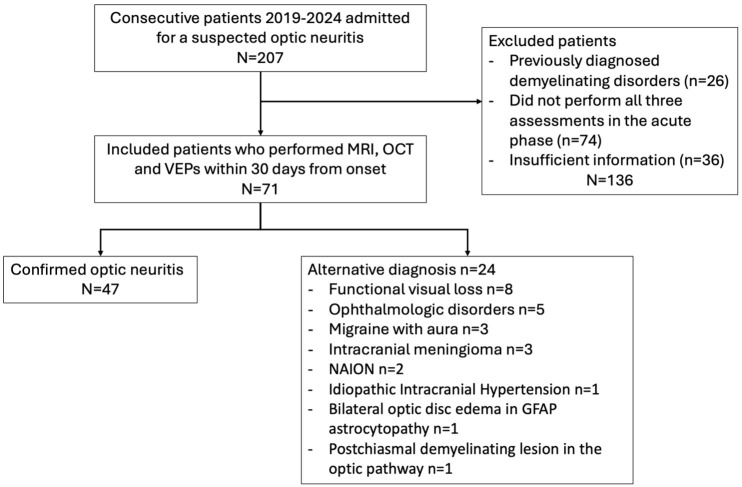
Study inclusion flowchart. GFAP: glial fibrillary acidic protein; MRI: magnetic resonance imaging; OCT: optical coherence tomography; NAION: non-arteritic ischemic optic neuropathy; VEP: visual evoked potentials.

We retrospectively screened consecutive patients admitted to the IRCCS Mondino Foundation between January 1 2019 and August 1 2024 with suspected ON. We only included patients who performed OCT, MRI, and VEPs within 30 days from symptoms onset. Patients were identified through a combined search of International Classification of Diseases (ICD) codes for ON (37730, 37732) and keyword searches (“optic neuritis,” “optic neuropathy,” “vision loss”) in referral notes and in examination queries. Patients with a previous diagnosis of a demyelinating disorder were excluded, as well as those with known ocular comorbidities potentially interfering with afferent visual system assessment (e.g. glaucoma, macular degeneration, or marked refractive errors). We collected data on the information requested to satisfy the clinical ICON criteria from medical reports. These included the presence of subacute visual loss, monocular or binocular involvement, orbital pain worsening on eye movements, dyschromatopsia, and the presence of RAPD. Acuity was assessed with Snellen charts and recorded in the decimal scale. A diagnosis of confirmed ON was made based on clinical and paraclinical evidence, exclusion of other causes and confirmed by two independent assessors with neuro-ophthalmology expertise (GG, ER). The two assessors evaluated each case independently and were blinded to each other’s diagnostic conclusion during their initial review. Only after completing their individual assessments did they meet to resolve discrepancies through consensus. Confirmed ON required: a suggestive clinical presentation and the positivity of at least one paraclinical test; exclusion of alternative diagnoses through history, examination, and ancillary tests performed during the admission; evaluation of steroid responsiveness degree and its pattern. Diagnosis of an underlying disorder where ON episodes have a characteristic phenotype (MS, MOGAD, AQP4-NMOSD) was also considered as a supportive feature. Patients with confirmed ON were ultimately grouped into four diagnostic categories according to published diagnostic criteria: MS-ON (MS-ON),^13^ MOG-ON,^3^ AQP4-ON,^14^ idiopathic ON (ION). A flowchart for study inclusion is represented in Figure 1.

The 2022 ICON criteria were applied to all patients in our cohort, after obtaining the data from all the paraclinical tests. As this is a retrospective study, only a diagnosis of “possible ON” was considered, consisting of at least one positive paraclinical test associated with a medical history suggestive of ON.^7^ The same cohort was then assessed for the same criteria by adding VEPs as a further paraclinical test.

### OCT

OCT was performed on a spectral-domain Cirrus HD-OCT (model 4000 version 5.0; Carl Zeiss Meditec), in a dark room, without pupil dilation. Peripapillary and macular data were obtained with the Optic Disk Cube 200 × 200 protocol and Macular Cube 512 × 128 protocol. OCT scanning was performed by experienced technicians, and scans were monitored to ensure fixation was reliable. Scans not meeting the OSCAR-IB consensus criteria were excluded.^15^ OCT alterations included, as suggested in the 2022 ICON criteria: a corresponding optic disk swelling acutely or an intereye difference in the macular ganglion cell inner plexiform layer (mGCIPL) of > 4% or > 4 μm or in the peripapillary retinal nerve fiber layer (pRNFL) of > 5% or > 5 μm.

### MRI

Participants were examined on a 1.5T MAGNETOM Sola or 3T MAGNETOM Skyra Siemens scanner (Siemens, Erlangen, Germany) using standard clinical protocols. MRI was considered suggestive of ON according to the parameters set out in the 2022 ICON criteria, which require either a hyperintense T2 lesion or contrast enhancement of the symptomatic optic nerve and sheaths acutely.

### Visual evoked potentials

VEPs were performed by checkerboard pattern reversal stimuli system (15’ and 30’ checks; mean luminance 50 cd/m^2^; maximum contrast, 100%; reversal rate 1 Hz) using a Mizar-Sirius device equipped with two dedicated software (Galileo NT and Basic BE), according to recommendations from the International Society for Clinical Electrophysiology of Vision (ISCEV) standard protocols.^16^ Scalp recordings were made with Ag/AgCl skin electrodes, 10 mm in diameter, placed accordingly to the 10–20 international system in Oz (active electrode), Cz (reference electrode), and at the Fpz (ground electrode).^17–19^ VEPs were recorded in response to 30’ (30’ VEP) and 15’ (15’ VEP) full-field, monocular pattern checks to obtain a prevalent activation of large (with 30’ checks) or small (with 15’ checks) axons. Two series of 100 artifact-free responses were averaged for each check and each eye. The latency to the peak of the major positive wave (P100) and the peak-to-peak amplitude were assessed and compared with the reference values in our laboratory (Supplementary Table 1). VEPs were considered abnormal if there was a P100 peak time delay after stimulation of a single eye or a significant intereye asymmetry in P100 peak time (including absent responses in the fellow eye). VEPs were performed by experienced electrophysiology technicians who monitored and corrected gaze fixation throughout the procedure. Even in cases of reduced visual acuity, fixation was facilitated through verbal guidance and continuous observation.

### Laboratory biomarker testing

MOG-IgG serum samples were tested using live CBAs for total MOG-IgG and for MOG-IgG1, as previously described.^20^ AQP4 testing was performed using a commercial AQP4 fixed CBA according to the manufacturer’s instructions (Euroimmun, Lubeck). CSF isoelectric focusing for OCBs was performed on agarose gel for paired serum and CSF specimens.

### Statistical analysis

Quantitative variables were reported as either mean (± standard deviation, SD) for continuous variables or medians (± interquartile range, IQR or full range) for discrete variables; categorical variables were reported as percentages. Subgroup comparisons were performed with parametric (χ2 test for qualitative or Student’s *t* for quantitative) or non-parametric (Fisher’s exact, Mann–Whitney) tests, as appropriate. Time from acute treatment start to test performance was compared through the ANOVA test, followed by Sidak-corrected post hoc comparisons between each group. Normality was assessed through the Shapiro–Wilk test. Statistical measures of diagnostic accuracy (sensitivity, specificity, positive predictive value [PPV], negative predictive value [NPV], accuracy) were calculated for OCT, MRI, MOG, and AQP4 antibodies and CSF OCB (which were grouped for the purpose of this analysis as laboratory biomarkers) and compared with those of VEPs. In addition, the same parameters were used to evaluate and compare the diagnostic performance of the 2022 ICON with and without the addition of VEPs. To evaluate the diagnostic performance of the paraclinical tests, receiver operating characteristic (ROC) curves were calculated for each test and plotted together to assess their area under the curve (AUC).

All analyses employed Stata/IC 14.0 for Mac (64-bit, StataCorp, College Station, Texas) and GraphPad Prism (V.9.0, GraphPad Software, La Jolla, California).

The project was approved by the Institutional Review Board of the IRCCS Policlinico San Matteo, Pavia (project code: 0020308/23). All patients included in the study provided their informed consent.

## Results

### Patients and clinical data

We initially identified 207 patients referred to our center for diagnostic workup of a suspected ON. We excluded 136 due to a previous demyelinating disorders diagnosis (*n* = 26) or incomplete assessment/insufficient clinical information (*n* = 110) (Figure 1). The final cohort included 71 patients. Mean age at onset was 36.3 (± 13.2) years, and 53 patients were females (75%). Clinical manifestations suggestive of ON included retro-orbital pain in 75% (53/71), dyschromatopsia in 63% (45/71), and RAPD in 37% (26/71). Median visual acuity at onset was 0.6 (range 0.0–0.10), and visual loss was predominantly unilateral (60/71, 85%).

Confirmed ON patients were 47/71 (66%) and fell into the following diagnostic groups: MS-ON (*n* = 36), ION (*n* = 4), MOGAD-ON (*n* = 6), and NMOSD-ON (*n* = 1). The remaining 24 patients had alternative diagnoses (Figure 1).

Patients with ON, compared to alternative diagnoses, had a higher frequency of clinical and paraclinical alterations suggestive of ON according to the ICON criteria, including retro-orbital pain, dyschromatopsia, RAPD, and a monocular vision loss (*p* < 0.001) (Table 1). Median visual loss at nadir was significantly lower in ON patients (0.5 [range 0.0–0.8] vs 1.0 [range 0.6–1.0], *p* < 0.001). All paraclinical tests were altered more often in patients with ON than those with other diagnoses (OCT, MRI, VEP all *p* > 0.001; Table 1).

**Table 1.** Clinical and paraclinical features of the study cohort.

	Whole cohort (*n* = 71)	Confirmed ON (*n* = 47)	Non-ON (*n* = 24)	*p*-value
Age, mean (SD)	36.29 (± 13.23)	36.5 (± 12.94)	39.91 (± 14.71)	0.78
Female sex, n%	53 (74%)	34 (71%)	20 (83%)	0.22
*Clinical features*				
Pain, n%	53 (75%)	46 (96%)	7 (29%)	< 0.001
Dyschromatopsia, n%	45 (63%)	43 (90%	2 (8%)	< 0.001
RAPD, n%	26 (37%)	25 (52%)	1 (4%)	< 0.001
Monocular visual loss, n%	60 (85%)	44 (92%)	16 (67%)	0.003
Nadir visual acuity, median (range)	0.6 (3–10)	0.5 (0.0–0.8)	1.0 (0.0–10)	< 0.001
*Paraclinical tests*				
OCT altered, n%	44 (62%)	38 (79%)	6 (25%)	< 0.001
Disk swelling, n%	18 (25%)	13 (27%)	5 (21%)	0.53
Intereye GCL, n%	27 (38%)	26 (54%)	1 (4%)	< 0.001
Intereye RNFL, n%	22 (31%)	19 (40%)	3 (13%)	0.02
MRI altered, n%	37 (52%)	34 (71%)	3 (13%)	< 0.001
T2 hyperintensity, n%	32 (45%)	31 (65%)	1 (4%)	< 0.001
Gd +, n%	18 (25%)	15 (31%)	3 (13%)	0.08
Positive biomarker, n%	33 (46%)	32 (67%)	1 (4%)	< 0.001
CSF OCB, n%	26 (37%)	25 (52%)	1 (4%)	< 0.001
AQP4 +, n%	1 (1%)	1 (2%)	0 (0%)	0.47
MOG +, n%	6 (8%)	6 (13%)	0 (0%)	0.07
VEP altered, n%	47 (66%)	45 (94%)	4 (17%)	< 0.001
Long P100, n%	42 (59%)	40 (83%)	3 (13%)	< 0.001
Low amplitude, n%	27 (38%)	24 (50%)	3 (13%)	0.002
IE asymmetry, n%	6 (8%)	3 (6%)	1 (4%)	0.35
1 positive test, n%	23 (32%)	18 (38%)	5 (21%)	0.14
> 1 positive test, n%	29 (41%)	27 (56%)	2 (8%)	< 0.001
No positive test, n%	19 (27%)	3 (6%)	17 (71%)	< 0.001

AQP4: aquaporin-4; CSF: cerebrospinal fluid; Gd: gadolinium; GCL: ganglion cell layer; IE: intereye; IQR: interquartile range; MOG: myelin oligodendrocyte glycoprotein; OCT: optical coherence tomography; ON: optic neuritis; RAPD: relative afferent pupil defect; RNFL: retinal nerve fiber layer; SD: standard deviation; VEP: visual evoked potentials.

### Paraclinical tests findings and diagnostic performance

**Figure 2.**
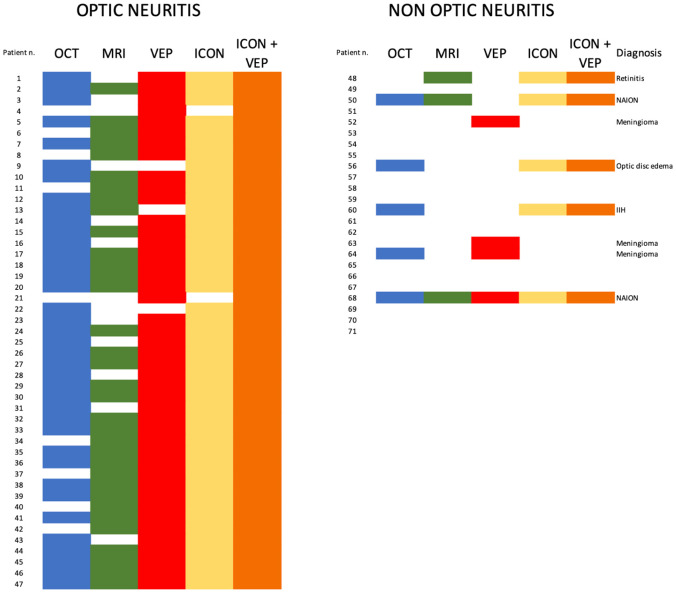
Visual representation of the study cohort. Each individual patient is a row; colored boxes represent positive paraclinical tests or a diagnosis of optic neuritis according to either the 2022 ICON criteria with or without VEPs. On the left side are patients with a confirmed diagnosis of optic neuritis; on the right are patients without optic neuritis. For incorrectly classified patients in the latter panel, detailed alternative diagnoses are provided. ICON: International Consortium of Optic Neuritis; IIH: idiopathic intracranial hypertension; MRI: Magnetic resonance imaging; OCT: optical coherence tomography NAION: non-arteritic ischemic optic neuropathy; VEP: visual evoked potentials.

The result of each diagnostic test for individual patients is summarized in Figure 2. VEPs showed alterations compatible with ON in 65% of included patients (46/71), being the most frequently altered paraclinical test. The most common VEP abnormality was an absolute increase in P100 latency (59%, 42/71), followed by a significant intereye asymmetry in P100 latency (8%, 6 patients). Twenty-eight patients had a reduced P100 peak amplitude (39%, 28/71), which is not considered specific for ON. OCT was altered in 62% of patients (44/71), and the most common abnormal finding was intereye mGCIPL difference (38%, 27/71) followed by intereye pRNFL difference (31%, 22/71) and optic disk swelling (25%, 18/71). MRI alterations suggestive of ON were found in 52% of patients (37/71) and included T2 optic nerve hyperintensity in 45% (32/71) and contrast enhancement of the symptomatic optic nerve and sheaths in 25% (18/71); 18% of patients (13/71) had both. Notably, only 45% of patients underwent specific orbit MRI protocols (32/71) with only 49% of MRI studies (35/71) including fat-suppressed sequences.

All patients underwent autoantibody testing. One was AQP4-IgG positive (diagnosed as NMOSD) and 6 were MOG-IgG positive (8%, diagnosed as MOGAD). Lumbar puncture was performed in 64/71 patients (90%), and 26/64 (37%) had unique-to-CSF OCBs. Patients were treated with high-dose intravenous corticosteroids (methylprednisolone 1000 mg); one MS and one NMOSD patient later underwent plasma exchange, which was performed after all diagnostic tests and did not affect their results. Time (measured in days) from acute phase treatment start to test performance was higher for MRI compared to both VEPs (1 [IQR 0–3] vs 0 [IQR 0–0], *p* = 0.01) and OCT (1 [IQR 0–3] vs 0 [IQR 0–0], *p* = 0.01).

**Figure 3.**
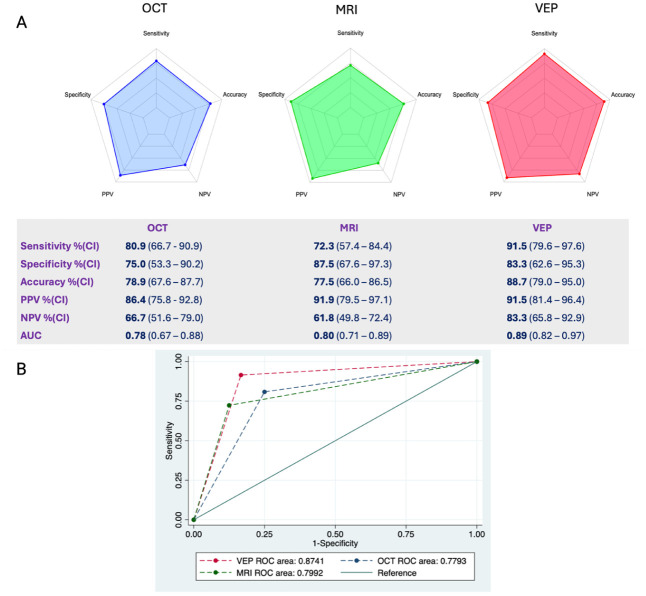
Diagnostic performance of paraclinical tests. Top: spider charts and diagnostic performance for individual diagnostic tests; diagnostic measures with confidence intervals are shown in the table. Bottom: comparison of receiver operating characteristic (ROC) curves for the three paraclinical tests. AUC: area under the curve; CI: confidence interval; MRI: Magnetic resonance imaging; OCT: optical coherence tomography; PPV: positive predictive value; NPV: negative predictive value; VEP: visual evoked potentials.

We then assessed the diagnostic performances of each paraclinical test, which are summarized in Figure 3, panel A. VEPs had the highest sensitivity (91.5%, CI:79.6–97.6) and accuracy (88.7%, CI: 79.0–95.0) compared to all other paraclinical tests. Coherently, VEPs had the highest AUC (0.89, CI: 0.82–0.97), followed by MRI (0.80, CI: 0.71–0.89) and OCT (0.78, CI: 0.67–0.88) (Figure 3, panel B). VEPs also had a specificity of 83.3% (CI: 62.6%–95.3%), higher than OCT (75%, CI: 53.3%–90.2%) but lower than MRI (87.5%, CI: 67.6%–97.3%). Specifically, 4 patients had a VEP alteration compatible with ON but an alternative diagnosis, namely a meningioma dislocating the optic nerve (*n* = 3) and non-arteritic ischemic optic neuropathy (NAION, *n* = 1). Biomarkers had the lowest sensitivity (68.1%, CI: 52.9%–80.9%) and overall accuracy (75.0%, CI: 62.60%–84.98%) although with very high specificity (94.1%, CI: 71.3%–99.8%).

**Figure 4.**
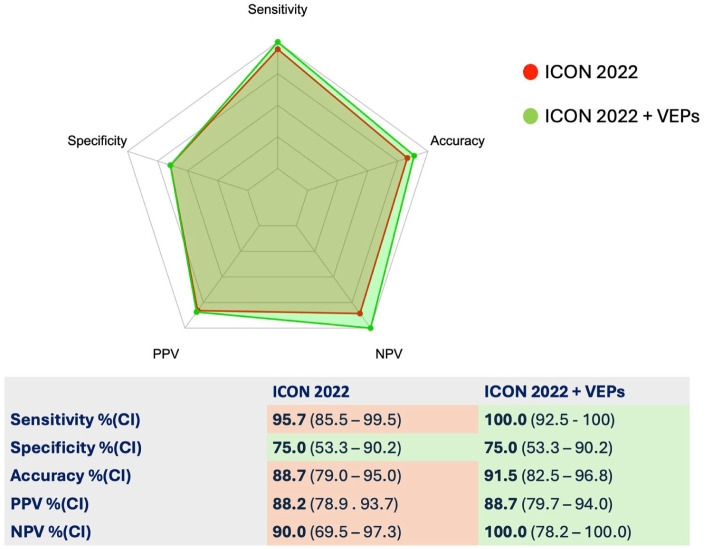
Diagnostic performance of the 2022 ICON criteria with and without the addition of VEPs. Diagnostic measures with confidence intervals are shown in the table. ICON: International Consortium of Optic Neuritis; AUC: area under the curve; CI: confidence interval; MRI: Magnetic resonance imaging; OCT: optical coherence tomography; PPV: positive predictive value; NPV: negative predictive value; VEP: visual evoked potentials.

We then retrospectively applied the 2022 ICON criteria to our cohort and compared their performance with the same criteria considering VEP as an additional paraclinical supporting feature (Figure 4 and Supplementary Table 2). The 2022 ICON criteria had an overall good performance, with a sensitivity of 95.7% (CI: 85.5–99.5), a specificity of 75.0% (CI: 53.3–90.2), PPV 88.2% (CI: 78.9–93.7), NPV 90.0% (CI: 69.5–97.3), and a global diagnostic accuracy of 88.7% (CI: 79.0–95.0). The addition of VEPs led to an improvement in sensitivity (100.0%, CI: 92.5–100.0) and NPV (100%, CI: 78.2–100.0) that resulted in an improvement in diagnostic accuracy to 91.5% (CI: 82.5–96.8) (Figure 4).

The two additional patients diagnosed as possible ON had a typical clinical history and suggestive VEP alterations. Presentation consisted of a subacute vision loss with retro-orbital pain and dyschromatopsia; both patients presented early in their disease course (3 and 5 days from symptoms onset, respectively) and visual loss was mild (visual acuity at nadir 0.8 and 0.7). Both patients showed good recovery after the administration of intravenous steroids, and one of them was later diagnosed with MS.

Six false-positive cases were identified, and these were the same in both the original 2022 ICON criteria and the version including VEPs. Two patients had MRI gadolinium enhancement of the optic nerve head, no CSF OCBs, an altitudinal visual field deficit and no response to steroid treatment and were ultimately diagnosed as NAION. One of these two also had a P100 latency increase. One patient with optic nerve head enhancement was diagnosed with pars plana retinitis (defined as pre-laminar ON according to the ICON criteria). One young girl with bilateral optic nerve head swelling on OCT was diagnosed with idiopathic intracranial hypertension (IIH) according to current diagnostic criteria.^21^ Another girl with bilateral optic nerve head swelling was positive for CSF glial fibrillary acidic protein (GFAP) antibodies and was diagnosed with GFAP astrocytopathy, in which papilledema without ON is a common feature.^22^ Finally, one patient with optic nerve head swelling had a post-traumatic intra-ocular infection.

Notably, 3 patients with a VEP alteration compatible with ON but an alternative diagnosis did not fulfill the ICON criteria as a clear alternative diagnosis to ON emerged from the paraclinical assessment, namely a meningioma dislocating the ON in all of them.

## Discussion

This study assessed the performance of VEPs in the diagnostic workup of ON, and their potential role as a further paraclinical test to consider in future iterations of the ICON diagnostic criteria. A strength of this work is the inclusion of a large, consecutive cohort of patients with and without ON, allowing the calculation of diagnostic performance measures in a real-life setting. Overall, the clinical phenotype of confirmed ON cases in our cohort (monocular involvement in 85%, pain in 75%, dyschromatopsia in 63%, and median visual acuity 0.6) is consistent with prior ON cohorts and supports the generalizability of our findings.

Our study showed that VEPs have good diagnostic performances for the evaluation of a suspected ON. Diagnostic measures and ROC values were the highest among paraclinical tests when each diagnostic tool was evaluated on its own. Coherently, retrospectively adding them to the 2022 ICON criteria improved sensitivity, PPV, NPV, and overall accuracy without hindering their specificity.

VEPs have been used for decades for ON assessment, where they are deemed of great diagnostic assistance.^10^ In line with this, their use has been incorporated into the 2024 McDonald criteria^23,24^ for the diagnosis of MS, where their alteration will be sufficient to establish the presence of an optic nerve lesion, and therefore to support the addition of the optic nerve as a fifth topography for the fulfillment of dissemination in space.^25,26^

A frequent concern regarding the inclusion of VEPs in the ICON ON criteria was their perceived lack of specificity. We suggest specifically looking for abnormalities that are highly related to inflammatory optic neuropathies, such as the delay in P100 wave both in absolute values and as a significant asymmetry compared with the fellow eye, alterations which have extensively been linked to ON.^8,12^ In some centers, VEPs can typically be performed earlier than MRI due to scanner availability. However, we acknowledge that this may vary significantly across institutions and health systems, because of logistical demands involved. Although VEPs interpretation can be challenging in patients with severe visual loss, experienced technicians can ensure adequate fixation, and an absent P100 response serves as a reliable pathological marker in accordance with ISCEV standards.

Our study confirmed the great specificity of MRI for a suspected ON evaluation. However, not all patients underwent dedicated optic nerve sequence imaging, and due to the broad timespan of study inclusion, earlier studies were performed with less sensitive sequences. Furthermore, as stated above, MRI was often performed later than other tests and after treatment start. This could be an explanation for the lower sensitivity of MRI alterations in our cohort. However, there is no indication for mandatory orbit MRI sequences to be acquired in the ICON criteria, and this reflects clinical practice, where orbital imaging is only reserved for selected cases and can be difficult to obtain due to scheduling constraints.

OCT testing can be very operator-dependent, especially when assessing imaging artifacts.^26^ Macular segmentation and GCL measurement can be challenging to interpret in patients with optic disk edema or other ophthalmic comorbidities such as macular degeneration,^27^ and GCL artifacts can be found in up to 40% of healthy subjects.^28^ However, the value of OCT imaging increases progressively, being able to better show intereye asymmetry in retinal thickness during the recovery phase.

This study has limitations. First, the reference standard to assess the performance of ICON criteria was the diagnosis established by two expert operators, similar to a previous study.^29^ Even though this is arbitrary, the false negative patients (i.e. suspected ON patients that did not fulfill the ICON criteria) all had clinical pictures highly suggestive of ON associated with a dramatic response to steroid treatment, making the ON diagnosis highly likely. Importantly, since the index test (i.e. VEPs) was included in the adjudication of a confirmed ON diagnosis by the reviewers, our results are at risk of incorporation bias, which might inflate VEPs sensitivity.^30^ We acknowledge this limitation, and we believe that prospective studies with predefined timing and blinded interpretation would be the ideal way to address this issue in the future. However, because all patients in the cohort underwent the same complete set of paraclinical investigations, any potential bias related to test incorporation would apply uniformly across the cohort, allowing meaningful comparison between the different diagnostic modalities. Two additional patients were classified as having ON based on VEP abnormalities, which represented the only positive paraclinical finding. These patients presented with a clinical picture consistent with mild ON. Visual impairment was subtle, and both patients sought medical attention very early after symptoms onset, possibly before detectable abnormalities could be captured by other diagnostic tests.

In our cohort, the frequency of documented RAPD was lower than expected. This likely reflects the fact that RAPD and dyschromatopsia were often assessed in the acute or emergency setting, where optimal standardized neuro-ophthalmic testing is rarely feasible—a limitation also reported in previous ON studies.^31^ Moreover, many patients did not undergo specific orbital MRI sequences, and this could have reduced MRI sensitivity performances. Furthermore, MRI was often performed after the first steroid doses (median 1 day after treatment start), whereas VEPs and OCT were generally obtained before or at treatment initiation. This temporal pattern may have reduced the sensitivity of MRI, since corticosteroids can attenuate contrast enhancement. Pattern electroretinography (PERG) was not routinely performed in our center, which prevented us from distinguishing retinal from post-retinal contributions to VEP abnormalities. This limitation may partly contribute to the non-specificity of VEP findings in conditions such as compressive or ischemic optic neuropathies.

In conclusion, our study supports the utility of VEPs as a diagnostic tool in ON assessment and their evaluations as a supportive test in future iterations of the ICON ON criteria. They could represent an optional supportive test that may be helpful in selected clinical contexts, depending on local availability and diagnostic workflow.

## Supplemental Material

sj-docx-1-msj-10.1177_13524585261424125 – Supplemental material for The supporting role of Visual Evoked Potentials for the diagnosis of Optic Neuritis within the 2022 ICON criteria Supplemental material, sj-docx-1-msj-10.1177_13524585261424125 for The supporting role of Visual Evoked Potentials for the diagnosis of Optic Neuritis within the 2022 ICON criteria by Giacomo Greco, Eleonora Rigoni, Francesco Masi, Giuseppe Cosentino, Massimiliano Todisco, Lara Ahmad, Eleonora Tavazzi, Eduardo Caverzasi, Anna Pichiecchio, Stefania Bianchi Marzoli, Michele Terzaghi, Elena Colombo and Matteo Gastaldi in Multiple Sclerosis Journal
