# Supplementary material for: The supporting role of Visual Evoked Potentials for the diagnosis of Optic Neuritis within the 2022 ICON criteria
Source: Mult Scler. 2026 Mar 30;32(5):465–74. doi: 10.1177/13524585261424125 (PMC13100327; doi:10.1177/13524585261424125)
Supplement: sj-docx-1-msj-10.1177_13524585261424125 – Supplemental material for The supporting role of Visual Evoked Potentials for the diagnosis of Optic Neuritis within the 2022 ICON criteria [file sj-docx-1-msj-10.1177_13524585261424125.docx]

**Supplementary material**

Supplementary table 1. Reference VEP values derived from an internal database of healthy controls aged 20-60 years. ms: milliseconds; OD: oculus dextrus; OS: oculus sinistrus.

| **30’ check size** | **P100 latency – upper limit (ms)** | **P100 latency, intereye asymmetry – upper limit (ms)** | **P100 amplitude – lower limit (ms)** |
| --- | --- | --- | --- |
| OD | 118,4 | 8,85 | 2,8 |
| OS | 117,6 | 8,85 | 2,8 |

| **15’ check size** | **P100 latency – upper limit (ms)** | **P100 latency, intereye asymmetry – upper limit (ms)** | **P100 amplitude – lower limit (ms)** |
| --- | --- | --- | --- |
| OD | 125,5 | 13,5 | 3,4 |
| OS | 127,8 | 13,5 | 3,3 |

Supplementary Table 2. Contingency tables for the application of the 2022 ICON criteria without (upper table) and with (bottom table) the addition of VEPs as a supportive test. ON: Optic Neuritis; VEPs: visual evoked potentials.

| No VEPs | **Confirmed ON** | **Non ON** |  |
| --- | --- | --- | --- |
| **Criteria +** | 45 | 7 | 52 |
| **Criteria -** | 2 | 17 | 19 |
|  | 47 | 25 | 71 |

| With VEPs | **Confirmed ON** | **Non ON** |  |
| --- | --- | --- | --- |
| **Criteria +** | 47 | 9 | 56 |
| **Criteria -** | 0 | 15 | 15 |
|  | 47 | 24 | 71 |
